# Dissertation on the Practice of Medicine

**Published:** 1849-09

**Authors:** 


### BIBLIOGRAPHICAL NOTICE.

*A Dissertation on the Practice of Medicine*; Containing an account of the causes, symptoms and treatment of diseases: and adapted to the use of Physicians and Families. By TOMLINSON FORT, M. D. Milledgeville, Ga.: 1849, 8 vo. pp. 740.

The author of this work has for many years held a conspicuous position in Georgia as a practitioner of large experience and skill, enjoying the confidence of the public, and the respect of his professional brethren. An American publication, presenting such claims, and devoted to practical medicine, should receive at our hands a more extended critical examination than we can bestow in the present instance. We must plead the exigencies of the times as an apology for noticing it more briefly than comports with our inclination.

The work is designed not for the Profession solely, but also for families. As regards the propriety of this two-fold adaptation, there will probably exist a difference of opinion among our readers. Many will deem a practical treatise on medicine designed for popular reading, to be of a questionable nature, and, without knowledge of the character and position of the author, some might be led to suspect that the objects of the publication were not strictly ethical. We have no doubt, not only from the high reputation of Dr. Fort as an honorable member of the Profession, but from the internal evidence of the book, that however mistaken he may be in the ends proposed, his motives are beyond reproach. While we are ready to admit that there is some cogency in the reasons which he assigns, we are disposed to doubt the policy of attempting to indoctrinate the general reader in the truths of practical medicine while there exists, almost universally, entire ignorance of those branches of science which are essential prerequisites to the study of pathology and therapeutics. We have always advocated making Physiology an element of general education, and introducing it as a branch of instruction not only in our academic institutions but in our common schools. We have formerly contributed some time and effort toward an end which appears to us highly desirable, by giving popular lectures on the subject. Something has been effected in this way; but this method of diffusing scientific information has become almost nugatory and disreputable, from the number and character of the peripatetic lecturers with whom the country is overrun. More especially with reference to phisiology, they who have professed to communicate its interesting truths to the public by oral teaching, have usually been persons whose attainments have been wholly insufficient for the undertaking, and whose aims, often, are other than belong to a true spirit of philosophy or philanthropy. We are aware that all members of the profession do not attach importance to the popular dissemination of information concerning the structure and functions of the organism. Some, indeed, maintain that its results would be worse than nugatory; that, agreeably to the maxim contained in the so frequently quoted expression: "A little learning is a dangerous thing," it is unwise to encourage what in many instances would amount to a mere smattering of physiological knowledge. We are not of this opinion. We do not entertain such apprehensions. Let even a portion of the intelligent members of a community become tolerably acquainted with the structure and functions of the human system, and, we are persuaded, the medical profession would be more esteemed, because medical science would be better appreciated, and hydra-headed quackery would be less rampant than it now is. If the honestly credulous men and women who now complacently adopt the various fantastic theories and dogmas propounded by dreamers and designers assuming to be medical reformers, were in a condition of mind to listen to advice, we would commend to them the study of physiology as the best safeguard against infatuation and deception. If this be a correct view of the case, it becomes a question which should bd seriously entertained by the profession, how is information of this kind to be diffused? If it be a matter of importance, not only to the medical profession, but to the interests of the public, that physiological knowledge be rendered popular, it is becoming that the profession deliberate and act accordingly. But we waive for the present the discussion of this topic; and, returning to the point which has led to this train of discursive remark, we must again express our distrust of the propriety of endeavoring to teach the public practical medicine, without previously providing for instruction in those fundamental branches which are considered essential for the medical student to prosecute, before he is qualified to understand the laws of disease, and the methods of their cure.

We proceed to say a few words of Dr. Fort's work as a treatise on practical medicine adapted to the professional reader. The author evidenly is an observing, sagacious practitioner, who, in the constant exercise of practical duties, has failed to keep himself fully posted up in medical literature. This, indeed, he acknowledges in his preface. Such men are apt, if not to over-estimate their own practical attainments, to un-derestimate the contributions by others which, collectively, render our science an art progressive, as it undoubtedly is. Dr. Fort's treatise is valuable as containing a host of original observations and suggestions, particularly pertaining to the treatment of diseases, but as a system of medicine it is deficient, especially in morbid anatomy, pathology and diagnosis, and could not be recommended to the student as a fair exponent of the present condition of the science. For example, under the head of Inflammation of the Lungs, he treats of Pneumonia and Pleurisy as different forms of the same disease, declaring that "the distinction between these two diseases, even under the stethescopic investigations of the day, is, to say the least of it, difficult. Nor do we derive, in a practical point of view, the benefit from these investigations which they at first appear to promise. The treatment of the disease in either form is about the same; and it would be vain to attempt to make the distinctions between them manifest to the inexperienced reader." This would be carrying us back to the thoracic pathology and diagnosis of the days of Cullen. What would be thought of the medical student on his examination for graduation at any of our medical colleges, who should be found unable to state the prominently distinctive marks, rational and physical, of the two diseases, which this author thus unites?

While we thus allude to the defects of the work, which justice to the present condition of medicine demands, we must express the satisfaction derived from an examination of it, as containing the views and experience of a sagacious, judicious senior practitioner. In the latter aspect the work is really valuable, and the author is richly entitled to the thanks of the profession for its publication. We trust that its reception will be such as to induce him to fulfil, in a future edition, the promise, which he makes, of remedying its defects, and doing justice to the labors of others.

